# Comment on: Comparison between thoracic epidural analgesia and rectus sheath catheter analgesia after open midline major abdominal surgery: randomized clinical trial

**DOI:** 10.1093/bjsopen/zrac101

**Published:** 2022-08-08

**Authors:** Matthew E Sinnott, Thomas P Heinink

**Affiliations:** Anaesthetics department, Frimley Park Hospital, Camberley, UK; Anaesthetics department, Frimley Park Hospital, Camberley, UK

*Dear Editor*

We read the study by Krige *et al.*^1^ with great interest and commend the authors for their well designed and pragmatic trial. We feel that the lack of any visceral component to even optimally placed rectus sheath catheters (RSCs) must be emphasized. A significant aspect of the quality of analgesia delivered by thoracic epidural analgesia (TEA) is the visceral component provided by local anaesthetic delivery directly to the neuraxis.

This lack of visceral analgesia is acknowledged by the authors as a reason for shorter time to first opioid in the RSC group. However, given that overall postoperative opioid consumption between groups was no different until day three, and efforts were taken to negate the impact of epidural fentanyl delivery via the administration of a fentanyl patch in the RSC group, it is perhaps unsurprising that the primary outcome favoured TEA.

The lack of inferiority in functional analgesia (arguably more important than single pain scores at specific points in time), alongside the reduced vasopressor requirements observed, and the implicit nursing burden associated with epidural analgesia, make RSCs an attractive option given the continued pressure on critical care capacity. Further work exploring these secondary outcomes in greater detail would therefore be very valuable. We congratulate the authors again for a thought-provoking study.
